# Evaluation of Medical Information on Male Sexual Dysfunction on Baidu Encyclopedia and Wikipedia: Comparative Study

**DOI:** 10.2196/37339

**Published:** 2022-08-09

**Authors:** Ming Ma, Saifu Yin, Mengli Zhu, Yu Fan, Xi Wen, Tao Lin, Turun Song

**Affiliations:** 1 Department of Urology and Organ Transplantation Center West China Hospital Sichuan University Chengdu, Sichuan Province China; 2 Core Facilities West China Hospital Sichuan University Chengdu, Sichuan Province China; 3 Department of Urology West China Hospital Sichuan University Chengdu, Sichuan Province China

**Keywords:** sexual dysfunction, digital health, Baidu Encyclopedia, Wikipedia, internet, health information, DISCERN instrument

## Abstract

**Background:**

Sexual dysfunction is a private set of disorders that may cause stigma for patients when discussing their private problems with doctors. They might also feel reluctant to initiate a face-to-face consultation. Internet searches are gradually becoming the first choice for people with sexual dysfunction to obtain health information. Globally, Wikipedia is the most popular and consulted validated encyclopedia website in the English-speaking world. Baidu Encyclopedia is becoming the dominant source in Chinese-speaking regions; however, the objectivity and readability of the content are yet to be evaluated.

**Objective:**

Hence, we aimed to evaluate the reliability, readability, and objectivity of male sexual dysfunction content on Wikipedia and Baidu Encyclopedia.

**Methods:**

The Chinese Baidu Encyclopedia and English Wikipedia were investigated. All possible synonymous and derivative keywords for the most common male sexual dysfunction, erectile dysfunction, premature ejaculation, and their most common complication, chronic prostatitis/chronic pelvic pain syndrome, were screened. Two doctors evaluated the articles on Chinese Baidu Encyclopedia and English Wikipedia. The Journal of the American Medical Association (JAMA) scoring system, DISCERN instrument, and Global Quality Score (GQS) were used to assess the quality of disease-related articles.

**Results:**

The total DISCERN scores (*P*=.002) and JAMA scores (*P*=.001) for Wikipedia were significantly higher than those of Baidu Encyclopedia; there was no statistical difference between the GQS scores (*P*=.31) for these websites. Specifically, the DISCERN Section 1 score (*P*<.001) for Wikipedia was significantly higher than that of Baidu Encyclopedia, while the differences between the DISCERN Section 2 and 3 scores (*P*=.14 and *P*=.17, respectively) were minor. Furthermore, Wikipedia had a higher proportion of high total DISCERN scores (*P*<.001) and DISCERN Section 1 scores (*P*<.001) than Baidu Encyclopedia. Baidu Encyclopedia and Wikipedia both had low DISCERN Section 2 and 3 scores (*P*=.49 and *P*=.99, respectively), and most of these scores were low quality.

**Conclusions:**

Wikipedia provides more reliable, higher quality, and more objective information than Baidu Encyclopedia. Yet, there are opportunities for both platforms to vastly improve their content quality. Moreover, both sites had similar poor quality content on treatment options. Joint efforts of physicians, physician associations, medical institutions, and internet platforms are needed to provide reliable, readable, and objective knowledge about diseases.

## Introduction

Knowledge regarding health and well-being is cobbled together from health care professionals, family, friends, books, newspapers, magazines, educational pamphlets, radio, television, and pharmaceutical advertisements [1]. However, we are increasingly heading online for answers rather than pursuing information through these other avenues [2]. Approximately 6% of all internet searches in the United States are health-related [3], and it is believed that internet searches have become people’s first choice of method to seek information regarding health issues [4]. In addition, the population of netizens in mainland China reached 1011 million in 2021, and the number of online medical users in China had reached 239 million by June 2021, accounting for 23.7% of total internet users [5]. Information quality, emotional support, and source credibility have significant and positive impact on the likelihood of health care information adoption, and among these factors, information quality has the biggest impact on patients’ adoption decisions [6]. Given the large amount of inaccurate information online, users are very easily misinformed [1]. Previous studies showed that the quality of online health information is problematic [7,8]. Thus, the assessment of source reputability and the veracity of information is a crucial and urgent task.

As the most common male sexual dysfunctions, erectile dysfunction (ED; the persistent inability to attain and maintain an erection sufficient to permit satisfactory sexual performance) and premature ejaculation (PE; poorly controlled and rapid ejaculation) greatly affect the quality of life of patients [9,10]. Furthermore, sexual dysfunction is closely associated with chronic prostatitis/chronic pelvic pain syndrome (CP/CPPS; urologic pain or discomfort in the pelvic region associated with lower urinary tract symptoms) and is the most common complication [11,12]. The prevalence of CP/CPPS in men is about 8.2%, and men with CP/CPPS are more prone to ED and PE than the general population [13]. A previous study found that nearly half of patients with a self-reported diagnosis of CP/CPPS reported mild to severe ED [14]. A meta-analysis of 24 studies suggested that the overall prevalence of sexual dysfunction in patients with CP/CPPS was 0.62 [15]. In particular, our previous study found that “prostate” and “prostatitis” were the most queried terms by Chinese users with PE [16], which highlighted the stigma and preferences of these patients [17]. In addition, the complex and unclear etiology of CP/CPPS and sexual dysfunction not only challenges clinicians in the choice of treatment but also seriously affects the quality of life of patients. Previously, public interest and the change over time in the search volume for sexual dysfunctions and lower urinary tract symptoms were analyzed [16,18,19]. People tended to consult Dr. Internet in a combined manner on these issues for treatment decision-making. Therefore, the issue of sexual dysfunction is commonly investigated with CP/CPPS.

Wikipedia, the most popular and consulted encyclopedia website in English, is a web-based encyclopedia that provides valuable web-based health information [20]. Previous studies have shown that Wikipedia is a reasonably reliable medical resource and it was ranked higher on search engines than other general websites [21,22]. Unfortunately, on May 19, 2015, “Chinese Wikipedia” announced that mainland Chinese servers would be shut down because of violation of mainland China’s laws due to the attack and destruction of the internet. As the equivalent Wikipedia for Chinese internet users, the Baidu platform and its Encyclopedia service is the most popular and frequently consulted encyclopedia site in mainland China [23,24]. In mainland China, with 766 million users actively using the Baidu search service, its usage in relation to health inquiries and symptom confirmation accounts for 66.83% of use, and health and medical topics ranked first among science topics [24]. Our previous research on the Baidu search index showed that the search demands by its users for sexual dysfunction and lower urinary tract symptoms are huge. However, users often get irrelevant online medical information, and there is little evaluation of the quality of Baidu-related content [16,19]. The purpose of this paper was to assess the reliability, readability, and objectivity of Wikipedia and Baidu Encyclopedia content on ED, PE, and CPPS/CP for the advancement of internet medicine.

## Methods

### Data Sources

The contents analyzed in this study are available on Chinese Baidu Encyclopedia and English Wikipedia. The Chinese Baidu Encyclopedia and English Wikipedia were investigated for articles on ICD-10 version 2016 codes. All possible synonymous and derivative keywords for each term were screened. Two doctors evaluated the articles on Chinese Baidu Encyclopedia and English Wikipedia. Any disagreement was reviewed by and arbitrated by a third reviewer who was an expert on sexual dysfunction. All authors have many years of experience in andrology and urology and are competent in the diagnosis and treatment of male sexual dysfunction and urinary disorders. These reviewers have professional knowledge of male sexual dysfunction and urinary disorders and can make professional evaluations.

### Assessment of the Quality of the Research Articles

The Journal of the American Medical Association (JAMA) scoring system [25], DISCERN instrument [26], and Global Quality Score (GQS) [27] were used to assess the quality of disease-related articles. The contents of these scoring tables are shown in Multimedia Appendix 1. The JAMA scoring system is a well-known tool for evaluating the quality of information obtained from health-related websites. It includes 4 evaluation dimensions: author, attribution, disclosure, and currency. If it meets the requirements of each dimension, it will get 1 point, and the deimension with the highest quality will get 4 points. The DISCERN instrument has been developed to judge the quality of written health information [26]. To more comprehensively determine the quality of information in the article, the DISCERN tool consists of 15 questions plus an overall quality rating, and each is scored on a scale from 1 to 5. The first section of the DISCERN instrument is commonly used to evaluate the quality of published information, and the second section focuses on the quality of treatment choices offered to patients. The total score can range from 16 to 80, where a score of 63 to 80 suggests excellent quality, 51 to 62 indicates good quality, 39 to 50 indicates fair quality, and 16 to 38 indicates poor quality [26]. Experienced health information users and providers can use the DISCERN instrument to distinguish between high-quality and low-quality publications, so as to promote the generation of high-quality, evidence-based patient information. The GQS is a 5-point Likert scale that can subjectively rate the overall quality of each reviewed website. In addition to evaluating the overall quality of the website, GQS also considers the flow and ease of use of each website [28].

### Statistical Analysis

All databases were constructed with Excel 2019 (Microsoft Corporation, Redmond, WA). The Shapiro-Wilk test was used to test the normality of the data. Descriptive analyses are reported as means and SDs for normally distributed variables. Medians and IQRs are reported for non-normally distributed variables. To ensure the quality of these scores, the intraclass correlation coefficient (ICC) was used to evaluate interobserver reliability. ICC values range from 0 (untrusted) to 1 (fully trusted), and any concordance values less than 0.75 were discussed by the research team to clarify the discrepancy. For nonparametric tests, the Mann-Whitney *U* test was conducted to test the significance of different ranks by using SPSS, version 22.0 (IBM Corp, Armonk, NY). The Fisher exact test was used to test the difference in the frequency distribution of DISCERN scores. We used Prism 8 for macOS, version 8.4.0 (455; GraphPad Software Inc, San Diego, CA) to conduct statistical analyses and create figures. For the statistical analysis, *P*<.05 was considered significant.

## Results

### Content Characteristics

We searched for “erectile dysfunction,” “premature ejaculation,” “chronic prostatitis/chronic pelvic pain syndrome,” and similar keywords on English Wikipedia and Chinese Baidu Encyclopedia. The search results are shown in Table 1. Wikipedia has only 1 entry for a disease, corresponding to a specific article. In Baidu Encyclopedia, a disease may have multiple entries and multiple articles. The information sources of these articles are different, and the number of views varies greatly. In Wikipedia, an article about a disease is constantly supplemented by different registered individuals. However, Baidu Encyclopedia's content providers are official organizations or unregistered individuals. Moreover, some of the recently updated articles in Baidu Encyclopedia show that the information is more often provided by organizations or institutions and is certified by experts. In addition, both Baidu and Wikipedia provide links to external information, including videos, articles, and images, while some links are unrelated advertisements. The latter especially appear in Baidu Encyclopedia. Furthermore, Wikipedia provides its own features for assessing the quality of articles, and all Wikipedia articles included in this study were rated as grade C.

**Table 1:** Characteristics of the search results from 2 online platforms.

Themes		Wikipedia	Baidu Encyclopedia	*P* value^a^
**Available entries, n**				
	CPPS/CP^b^	2	3	N/A^c^
	ED^d^	1	2	
	PE^e^	1	3	
**Real-time updates, n**				
	Yes	4	8	.99
	No	0	0	
**External links, n**				
	Yes	4	6	.52
	No	0	2	
**Advertisement, n**				
	Yes	0	4	.21
	No	4	4	
**Author type, n**				
	Organization	4	5	.49
	Individuals	0	3	
Page views (x1000), median (IQR)		1673.2 (240.0-3878.9)	4119.7 (775.3-22029.8)	.37
Number of references, median (IQR)		53.5 (19.3-84.0)	0 (0.0-0.8)	.002

^a^A Mann-Whitney *U* test was conducted to test the significance of different ranks.

^b^CP/CPPS: chronic prostatitis/chronic pelvic pain syndrome.

^c^N/A: not applicable.

^d^ED: erectile dysfunction.

^e^PE, premature ejaculation.

### Overall Scores for Baidu Encyclopedia and Wikipedia

A 2-way mixed/random effects model was used to analyze the consistency of the ratings by the 2 independent reviewers. The ICC results showed good consistency between the 2 reviewers for the GQS scores (ICC=0.87), JAMA scores (ICC=0.91), and DISCERN scores (ICC=0.82).

**Figure 1:**
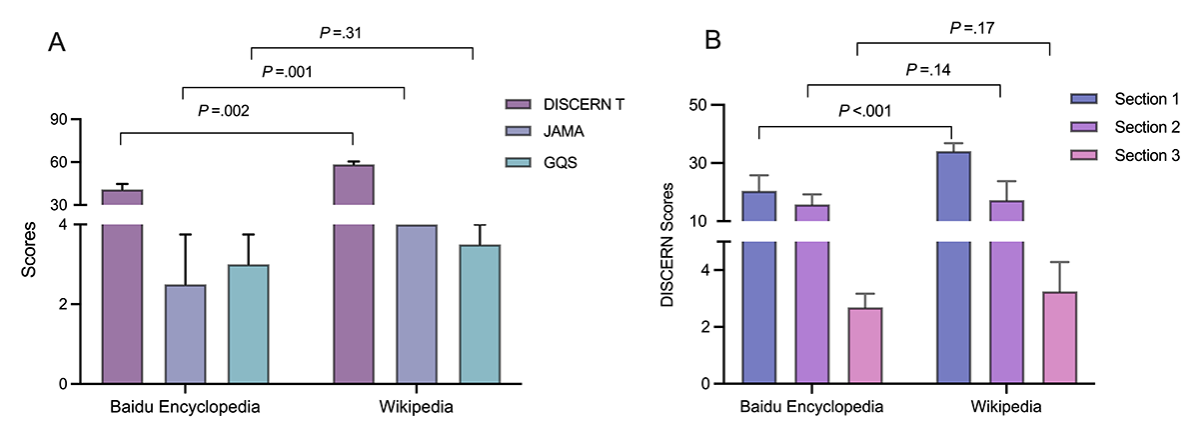
Overall comparison between Baidu Encyclopedia and Wikipedia: (A) median and IQR for DISCERN total scores, Journal of the American Medical Association (JAMA) scoring system scores, and Global Quality Score (GQS) scores; (B) median and IQR for the 3 DISCERN sections.

Comprehensively, the scores for Wikipedia were higher than those for Baidu Encyclopedia (Figure 1A). The contents in Wikipedia were significantly higher rated by the DISCERN tool and JAMA tool than those in Baidu Encyclopedia, suggesting that Wikipedia provides higher quality information. Although there was no statistical difference between the GQS scores for these websites, a numerically higher score on Wikipedia indicates that Wikipedia may provide better reading fluency and ease of use. In order to distinguish the differences between the 2 websites in more detail, we compared the DISCERN section scores for Baidu Encyclopedia and Wikipedia (Figure 1B). The DISCERN Section 1 score for Wikipedia was significantly higher than that for Baidu Encyclopedia, suggesting that Wikipedia provides more reliable and more objective information. The DISCERN Section 2 evaluates “How good is the quality of information regarding treatment choices?” There was no statistical difference between the DISCERN Section 2 scores for these websites, suggesting that they may have a similar impact on patients’ choice of treatment options. Section 3 is the overall rating of the publication, and the lack of statistical difference revealed that the overall quality of the publication as a source of information about treatment choices was similar for these websites.

### Overall Quality Comparison Between Wikipedia and Baidu Encyclopedia for the Theme of ED

**Figure 2:**
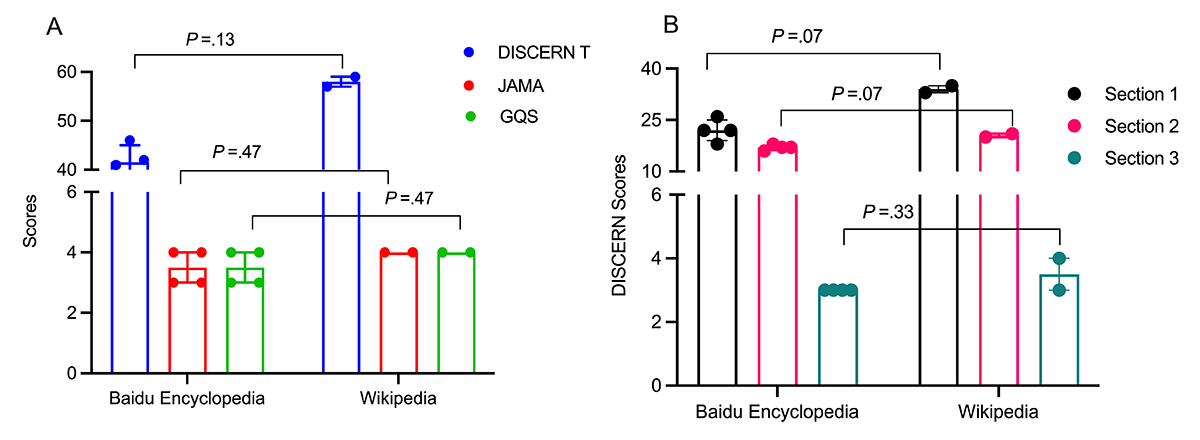
Comparison of erectile dysfunction (ED) scores between Baidu Encyclopedia and Wikipedia: (A) median and IQR for total DISCERN scores, Journal of the American Medical Association (JAMA) scoring system scores, and Global Quality Score (GQS); (B) median and IQR for the 3 DISCERN sections.

ED is one of the most common male sexual dysfunctions. By comparing the content scores for ED articles on Baidu Encyclopedia and Wikipedia, Wikipedia appeared to have numerically higher total DISCERN scores, JAMA scores, and GQS scores, but there were no statistically significant differences (Figure 2A). Furthermore, the 3 DISCERN section scores for Baidu and Wikipedia were also compared separately (Figure 2B). Wikipedia appeared to have numerically higher DISCERN Section 1 and 2 scores. In addition, they had similar DISCERN Section 3 scores. These results suggest that there is no statistically significant difference between Wikipedia and Baidu Encyclopedia scores for ED content.

### Overall Quality Comparison Between Wikipedia and Baidu Encyclopedia for the Theme of PE

**Figure 3:**
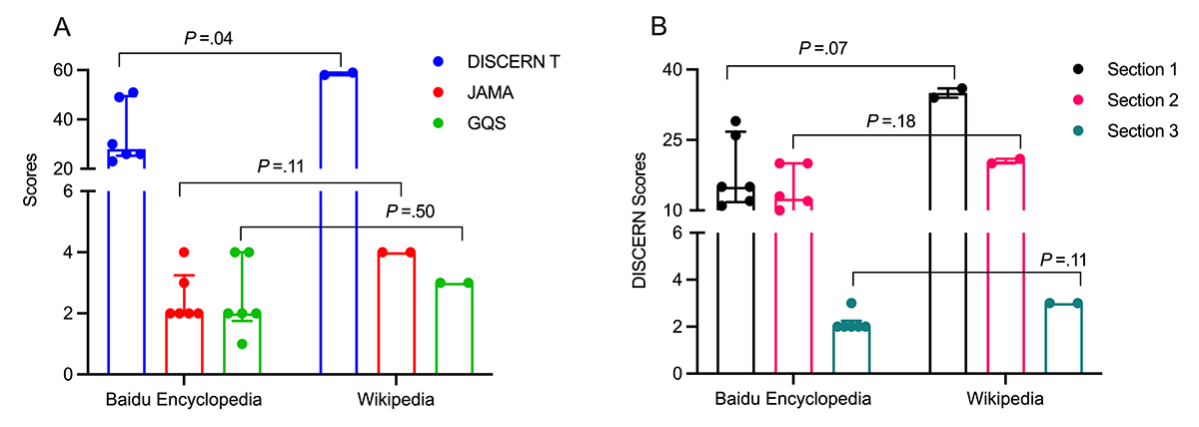
Comparison of premature ejaculation (PE) scores between Baidu Encyclopedia and Wikipedia: (A) median and IQR for total DISCERN scores, Journal of the American Medical Association (JAMA) scoring system scores, and Global Quality Score (GQS); (B) median and IQR for the 3 DISCERN sections.

A comparison of the scores for PE, the other most common sexual dysfunction disorder, showed that Wikipedia had a significantly higher total DISCERN score than Baidu Encyclopedia (Figure 3A). Although Wikipedia seemed to have higher JAMA and GQS scores than Baidu Encyclopedia (Figure 3A), this difference was not statistically significant, and all DISCERN section scores showed a similar trend (Figure 3B), which may be related to the great intragroup variability of Baidu Encyclopedia.

### Overall Quality Comparison Between Wikipedia and Baidu Encyclopedia for the Theme of CP/CPPS

**Figure 4:**
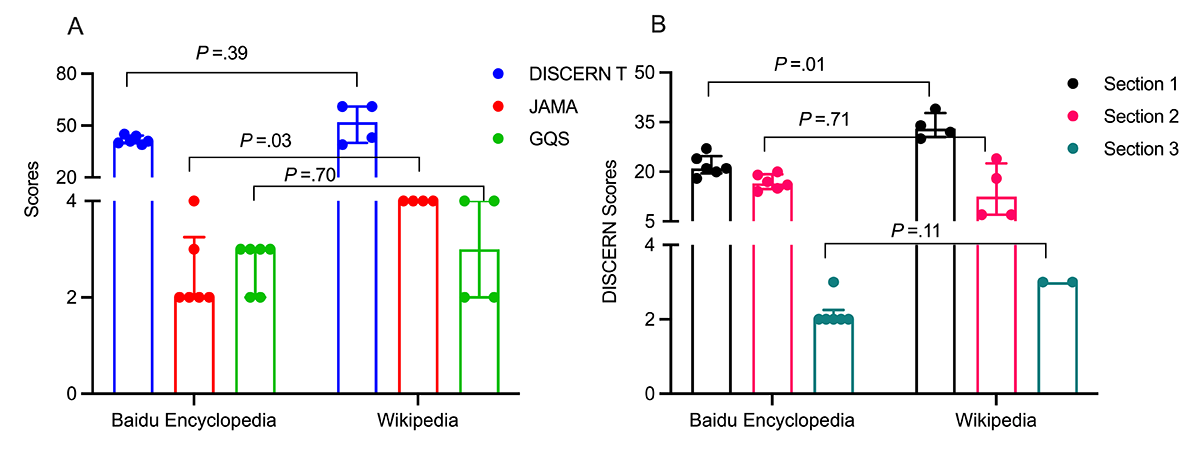
Comparison of chronic prostatitis/chronic pelvic pain syndrome (CP/CPPS) scores between Baidu Encyclopedia and Wikipedia: (A) median and IQR for total DISCERN scores, Journal of the American Medical Association (JAMA) scoring system scores, and Global Quality Score (GQS); (B) median and IQR for the 3 DISCERN sections.

CP/CPPS, as one of the most common concomitant diseases of sexual dysfunction, seriously affects the quality of life of male patients. By comparing the overall scores for Wikipedia and Baidu encyclopedia on CP/CPPS, we found that the scores of Baidu Encyclopedia were mostly fair quality, while the scores of Wikipedia ranged from fair quality to good quality (Figure 4A). Meanwhile, Wikipedia showed statistically higher JAMA scores, but there were no statistical differences between total DISCERN scores and GQS scores (Figure 4A). Furthermore, the DISCERN Section 1 score for Wikipedia was statistically significantly higher than that of Baidu Encyclopedia, while the DISCERN Section 2 and 3 scores for both sites were not significantly different from each other (Figure 4B).

### Distribution of the DISCERN Scores

After comparing the overall quality of the information for different diseases on Baidu encyclopedia and Wikipedia, the overall scores for Wikipedia seemed to be higher than those of Baidu encyclopedia, but some scores only showed numerical differences without statistical significance. Nevertheless, the differences in the distribution of scores that had numerical differences were seemingly obvious. Therefore, we performed further statistical analyses of the score distributions for Wikipedia and Baidu Encyclopedia. As aforementioned, according to the DISCERN standard, a total DISCERN score <50 (near 60%) is fair or poor quality, while a score >50 is good or excellent quality [26]. Based on this rule, we took a score of 3 for each question as the cutoff value; that is, a score higher than 3 points was defined as good quality.

The score distributions for each disease are shown in Table 2. Wikipedia had a higher proportion of total DISCERN and Section 1 scores distributed above 3 points, whether compared with the overall score or the score for each disease, and was significantly better than Baidu Encyclopedia. However, Baidu Encyclopedia and Wikipedia had low Section 2 and 3 scores, and most of these scores were ≤3, which are defined as low quality.

**Table 2:** Distribution of the DISCERN scores for each disease and comparisons via the Fisher exact test.

DISCERN		Overall			CP/CPPS^a^			ED^b^			PE^c^		
		Wikipedia, n (%)	Baidu Encyclopedia, n (%)	*P* value	Wikipedia, n (%)	Baidu Encyclopedia, n (%)	*P* value	Wikipedia, n (%)	Baidu Encyclopedia, n (%)	*P* value	Wikipedia, n (%)	Baidu Encyclopedia, n (%)	*P* value
**Total**													
	>3	38 (59.4)^d^	25 (19.5)^e^	<.001	17 (53.1)^f^	10 (20.8)^g^	.004	10 (62.5)^h^	7 (21.9)^f^	<.001	11 (68.8)^h^	8 (16.7)^g^	<.001
	≤3	26 (40.6)^d^	103 (80.5)^e^		15 (46.9)^f^	38 (79.2)^g^		6 (37.5)^h^	25 (78.1)^f^		5 (31.2)^h^	40 (83.3)^g^	
**Section 1**													
	>3	31 (96.9)^f^	19 (29.7)^d^	<.001	15 (93.8)^h^	7 (29.2)^i^	<.001	8 (100)^j^	6 (37.5)^h^	.006	8 (100)^j^	6 (25.0)^i^	<.001
	≤3	1 (3.1)^f^	45 (70.3)^d^		1 (6.2)^h^	17 (70.8)^i^		0 (0)^j^	10 (62.5)^h^		0 (0)^j^	18 (75.0)^i^	
**Section 2**													
	>3	5 (17.9)^k^	6 (10.7)^l^	.49	1 (7.1)^m^	3 (14.3)^n^	.64	1 (14.3)^o^	1 (7.1)^m^	.99	3 (42.9)^o^	2 (9.5)^n^	.08
	≤3	23 (82.1)^k^	50 (89.3)^l^		13 (92.9)^m^	18 (85.7)^n^		6 (85.7)^o^	13 (92.9)^m^		4 (57.1)^o^	19 (90.5)^n^	
**Section 3**													
	>3	2/4 (50.0)^p^	3 (37.5)^j^	.99	1 (50.0)^q^	0 (0)^r^	.40	1 (100)^s^	0 (0)^q^	.33	0 (0)^s^	0 (0)^r^	.99
	≤3	2 (50.0)^p^	5 (62.5)^j^		1 (50.0)^q^	3 (100)^r^		0 (0)^s^	2 (100)^q^		1 (100)^s^	3 (100)^r^	

^a^CP/CPPS: chronic prostatitis/chronic pelvic pain syndrome.

^b^ED: erectile dysfunction.

^c^PE: premature ejaculation.

^d^n=64.

^e^n=128.

^f^n=32.

^g^n=48.

^h^n=16.

^i^n=24.

^j^n=8.

^k^n=28.

^l^n=56.

^m^n=14.

^n^n=21.

^o^n=7.

^p^n=4.

^q^n=2.

^R^n=3.

^S^n=1.

## Discussion

### Principal Findings

Internet-based information is playing an increasingly important role in the diagnosis and treatment of patients, especially for privacy-sensitive conditions such as sexual dysfunction and related concomitant diseases. Comprehensive and objective information can help patients understand their condition, choose the right time to visit a doctor, and then improve their prognosis. However, incorrect or incomplete information may leave patients vulnerable to misdiagnosis, leading to delays in treatment and considerable health risks [1]. As a consequence, at a time when internet health care is booming, there is an urgent need to evaluate the credibility, readability, and accuracy of online resources. This study evaluated the reliability, readability, and objectivity of Baidu Encyclopedia and Wikipedia in terms of ED, PE, and CP/CPPS content. Overall, the total DISCERN scores and DISCERN Section 1 scores for the content provided by Wikipedia were significantly higher than those of Baidu Encyclopedia. Also, Wikipedia had a higher proportion of total DISCERN and Section 1 scores distributed within the high-quality range than Baidu Encyclopedia. Combined with higher JAMA scores, the results suggest that Wikipedia provided more reliable, higher quality, and more objective information than Baidu Encyclopedia. Baidu Encyclopedia and Wikipedia had low DISCERN Section 2 and 3 scores, and most of these scores were low quality. Similar DISCERN Section 2 and 3 scores for Wikipedia and Baidu Encyclopedia indicated that they had an analogic and mediocre impact on patients’ choice of treatment options. Although not statistically different, Wikipedia had numerically higher GQS scores, suggesting that Wikipedia might provide relatively better flow and be easier to use.

By June 2021, the number of online medical users in China was 239.33 million, and the utilization rate of the internet was 23.7%, an increase of 11.4% over December 2020 [29]. In an analysis of internet search trends in China, some scholars found that only 43.74% of the search results for PE were related to PE [16]. In another study on lower urinary tract symptoms, 1.13%-93.92% of the retrieved content was found to be irrelevant to lower urinary tract symptoms [19]. The study also found similar problems in the contents about these diseases in Wikipedia and Baidu Encyclopedia. Wikipedia provides more standardized and unified content, with standard templates for almost every disease, which allows readers to find the information they need quickly and accurately [30]. In contrast, the quality of content provided by Baidu Encyclopedia varies widely, with some recently updated articles providing more comprehensive content than Wikipedia, but the overall trend is a lack of standardization and formality. In Baidu Encyclopedia, the same disease may correspond to multiple entries and corresponding articles, which compare poorly with each other, and different articles may provide users with contradictory information, which can cause great confusion to users. The diversity of the content formats presented by Baidu Encyclopedia is consistent with the great variability of its overall score. The total DISCERN scores and JAMA scores for Wikipedia were significantly higher than those for Baidu Encyclopedia, and the proportion of Wikipedia scores within the high-quality distribution was also higher than those for Baidu Encyclopedia. These results suggest that Wikipedia provides higher quality information than Baidu Encyclopedia. In addition to the lack of a standard content presentation format, the low quality of Baidu Encyclopedia is also related to other features of its website, such as information sources and references. The contents of Baidu Encyclopedia are mostly sourced from official organizations or unregistered individuals, while information on Wikipedia is provided by registered users. The comparison shows that the quality of contents provided by unregistered individuals is always rated as “poor quality.” Accurate citation of high-quality references is an important guarantee for the reliability of a paper [31]. The contents provided by these unregistered individual users are almost always without references and extended information. By contrast, the quality of contents provided by registered users or official organizations are almost rated as “good quality,” with accurate references. These characteristics of the website are closely related to DISCERN Section 1 scores, and significantly higher DISCERN Section 1 scores for Wikipedia indicate that its publications are more reliable than those of Baidu Encyclopedia. The other 2 main focuses of the quality assessment are “How good is the quality of information regarding treatment choices?” and “the overall quality of the publication as a source of information about treatment choices.” Similar scores on DISCERN Sections 2 and 3 for Wikipedia and Baidu Encyclopedia indicated that they had an analogic and mediocre impact on patients’ choice of treatment options. Recent updates to Baidu Encyclopedia also show an increasing number of medical professionals involved in reviewing or writing the content, also significantly improving the DISCERN and JAMA scores. This comparison suggests that the inconsistency of disease presentation formats and differences in information sources may account for the lower Baidu scores.

CP/CPPS is characterized by localized pain or discomfort in the abdomen, pelvis, and genitals, usually with lower urinary tract symptoms, psychosocial disorders, and sexual dysfunction [11,12]. The relationship between sexual dysfunctions and CP/CPPS has been studied more extensively [32]. Previous studies have shown a good correlation between the severity of symptom scores between the 2 clinical conditions, CP and PE, and that approximately 49% of male patients with CP have concomitant sexual dysfunction [33]. It addition, “prostate” and “prostatitis” were the most queried terms by Chinese users with PE [16]. The complex and heterogeneous pathophysiology of CP/CPPS makes the management of this troublesome situation very challenging both for clinicians and patients, and approximately 50% of older patients experience recurrence [34]. The UPOINT System classifies CP/CPPS patients into 7 different subgroups based on symptoms: urologic, psychosocial, organ-specific, infectious, neurologic, tenderness (pelvic floor tenderness), and sexual dysfunction; then, it proposes specific treatment plans based on the different subgroups [35]. There is growing evidence that the addition of second-line therapies, such as 5-phosphodiesterase inhibitors, antidepressants and muscle relaxants, according to the UPOINT System approach, can significantly improve patients’ CP/CPPS symptoms [36]. These results showed that CP/CPPS and sexual dysfunctions can directly or indirectly increase the economic burden of health care and seriously affect patients’ quality of life. Patients with CP/CPPS or sexual dysfunction may feel too embarrassed to discuss their problems with doctors due to the influence of the Chinese culture, and they are likely more willing to look for disease-related information, such as symptoms, diagnosis, treatment methods, prognosis, and hospital rankings, on the internet first. There is no doubt that the information these patients access from the internet affects their perception of their health status, which in turn affects treatment choices and disease prognosis.

By comparing the contents for ED, PE, and CP/CPPS on Baidu Encyclopedia and Wikipedia, we found that the consistency of Wikipedia is better, with almost all content rated as “good quality,” while the scores for Baidu Encyclopedia were mostly “fair quality.” Take PE-related articles in Baidu Encyclopedia as examples. Both reviewers rated “早发性射精” (early-onset ejaculation) as “poor quality.” After analyzing the content on the web page for “early-onset ejaculation,” we found there was no introduction to “examination, diagnosis, and treatment,” and the content in the article was not objective and scientific. Contrary to the lack of effective information, there are more than 25 irrelevant advertising links and only one reference on this web page. The content on the “早泄” (premature ejaculation) page on Baidu Encyclopedia was rated as “good quality,” and the information was more comprehensive and objective than that for “early-onset ejaculation.” Corresponding to the quality grades for “early-onset ejaculation” and “premature ejaculation,” there was a huge difference in page views (early-onset ejaculation/premature ejaculation: 33,506/25,747,398). The discrepancy may be related to the inconsistent identity of content providers. The irrelevant advertising links or misleading information obtained by users using Baidu Encyclopedia may be related to the fee-based editing service. There are many third-party underground industries that charge fees to write Baidu Encyclopedia entries on their behalf, so as to insert advertisements and achieve the purpose of attracting patients. In order to improve the quality of the health information, Baidu Encyclopedia announced the “rainbow plan” on December 9, 2012, wherein all medical entries could only be edited and revised by certified medical experts [37]. This is consistent with the findings of this study that an increasing number of medical professionals are involved in reviewing or writing content for Baidu Encyclopedia. Consequently, attracting, encouraging, and even recruiting more medical professionals to draft or proofread the content about disease presentation provided on these websites may ensure the content is objective and comprehensive. At the same time, the Baidu Encyclopedia platform should strengthen content regulation and establish a review mechanism to remove interest-related content.

In contrast, Wikipedia has its own content quality evaluation system, such as the “Wiki-Project article quality grading scheme” and the “Wiki-Project priority assessments” [38,39]. In this study, all included Wikipedia articles were rated as grade C, which means “Useful to a casual reader, but would not provide a complete picture for even a moderately detailed study” and “Considerable editing is needed to close gaps in content and solve cleanup problems.” The “Wiki grading” for these Wikipedia articles is similar to the grading by the 3 grading tools applied in this paper. That is, the quality of these Wikipedia articles is almost “good quality” but far from “excellent quality,” and all articles needed further improvement. Despite this fact, the formality and drafting on Wikipedia are better because of the clear attribution and disclosure it provides. As mentioned earlier, there is a lack of uniform standards for writing Baidu Encyclopedia content, many of the information sources are not supported by academic references, and external links are mostly related to advertisements. Hence, though the content on both sites leaves much to be desired, as a source to popularize science, the content on Wikipedia could at least guide interested individuals to the right source of informations, while Baidu Encyclopedia is more likely to provide misleading information.

In the era of rapid internet development, more patients have started to try online consultations [40]. This change in mode of treatment has presented new opportunities and challenges for doctors, medical institutions, physician associations, internet platforms, and patients. In this study, we evaluated the objectivity, reliability, and readability of the content on sexual dysfunction and CP/CPPS on Baidu and Wikipedia and found that the quality of the content provided by both sites was not “excellent quality” and needed to be improved. This study is only a microcosm of the vast amount of information available in internet-based health care. Considering the increasing coverage of the internet, more users will be influenced by internet-based information, and incorrect or incomplete information will have a negative impact on users’ decision-making. Therefore, we believe that, in the era of the internet information explosion, physicians, physician associations, and medical institutions should make full use of their expertise and become more involved in the construction of internet-based health care by providing objective and comprehensive content. Internet platforms, on the other hand, should strengthen the regulation and review of medical-related content and remove false or irrelevant content. Wikipedia already has a relatively complete self-censorship system and self-evaluation system, but Baidu Encyclopedia has almost no achievements in this regard. In China, the country with the world’s largest population, the importance of popular science education for the whole society and the world is self-evident. Baidu Encyclopedia, as the largest platform for online science education in China, still needs to be greatly enhanced to take up the corresponding social responsibility. Through the joint efforts of physicians and the platform, we hope to achieve the goal of providing users with timely access to correct, objective, comprehensive, and valid information when seeking medical advice or searching for health science content on the internet.

### Limitations

Some limitations must be addressed in this study. This study only presents the results of medical professionals’ evaluations of health-related science content on the internet, and further research is needed on the specific impact of this information on the audience and readers. Since information on the internet is updated quickly, there may be some bias between the study results and the actual situation, and the data need to be updated in real time to ensure that the findings are true and valid. In addition, the difference in the number of Chinese and English entries indicates the information received by users will be significantly different because of the entries they choose to click. Therefore, our “combined” evaluation cannot fully represent the quality of the information they really receive. Fortunately, with the availability of infodemiology research, academics can combine content analysis and infodemiology search trends to better elucidate the impact of health-related information on the internet on users, society, and the health care industry.

### Conclusions

Internet medicine, as a new medical model in the new era, provides strong support for users to understand disease information and choose the timing of treatment in a timely manner. Although it is more formally composited, Wikipedia also provides more reliable, higher quality, and more objective information than Baidu Encyclopedia. They also have a similar impact on patients’ choice of treatment options, and the websites are similar in terms of flow and ease of use. To promote the healthy and sustainable development of internet health care, the joint efforts of physicians, physician associations, medical institutions, and internet platforms are needed to provide more reliable, accessible, and comprehensible disease knowledge to the public.

## Appendix

Multimedia Appendix 1 Scoring tables for the Journal of the American Medical Association (JAMA) scoring system, DISCERN instrument, and Global Quality Score (GQS).

## Abbreviations

CP/CPPS: chronic prostatitis/chronic pelvic pain syndrome
ED: erectile dysfunction
GQS: Global Quality Score
ICC: intraclass correlation coefficient
JAMA: Journal of the American Medical Association
PE: premature ejaculation
