# Supplementary material for: Evaluation of Medical Information on Male Sexual Dysfunction on Baidu Encyclopedia and Wikipedia: Comparative Study
Source: J Med Internet Res. 2022 Aug 9;24(8):e37339. doi: 10.2196/37339 (PMC9399830; doi:10.2196/37339)
Supplement: Multimedia Appendix 1 [file jmir_v24i8e37339_app1.pdf]

**Basic information**	
Reviewer	Name: ________________
Searching Themes	Keywords:
Search engine	□ Wikipedia □ Baidu Encyclopedia
Access date	Access date: ________________
Available entries	1. http://<br>2. ……<br>3. ……<br>4. ……<br>……
Selected entry	
Real-time Update	□ Yes □ No
Author type	□ Organization □ Individuals
Page views	
Reference number	
External links	□ Yes □ No
Advertisement	□ Yes □ No
**Assessment of the Quality of the Research Articles**	
**JAMA**	□ Authorship<br>Authors and contributors, their affiliations, and relevant credentials should be provided
	□ Attribution<br>References and sources for all content should be listed clearly, and all relevant copyright information should be noted
	□ Disclosure<br>Website "ownership" should be prominently and fully disclosed, as should any sponsorship, advertising, underwriting, commercial funding arrangements or support, or potential conflicts of interest
	□ Currency<br>Dates when content was posted and updated should be indicated
**JAMA scores: _______**	
**GQS**	1. Poor quality, very unlikely to be of any use to patients
	2. Poor quality but some information present, of very limited use to patients
	3. Suboptimal flow, some information covered but important topics missing, somewhat useful to patients
	4. Good quality and flow, most important topics covered, useful to patients
	5. Excellent quality and flow, highly useful to patients
**GQS scores: _______**	
**DISCERN scores**	**Section 1 Is the publication reliable?**
□ 1 □ 2 □ 3 □ 4 □ 5	1. Are the aims clear?
□ 1 □ 2 □ 3 □ 4 □ 5	2. Does it achieve its aims?
□ 1 □ 2 □ 3 □ 4 □ 5	3. Is it relevant?
□ 1 □ 2 □ 3 □ 4 □ 5	4. Is it clear what sources of information were used to compile the publication (other than the author or producer)?
□ 1 □ 2 □ 3 □ 4 □ 5	5. Is it clear when the information used or reported in the publication was produced?
□ 1 □ 2 □ 3 □ 4 □ 5	6. Is it balanced and unbiased?
□ 1 □ 2 □ 3 □ 4 □ 5	7. Does it provide details of additional sources of support and information?
□ 1 □ 2 □ 3 □ 4 □ 5	8. Does it refer to areas of uncertainty?
**Section 1 scores: ______**	

	**Section 2 How good is the quality of information regarding treatment choices?**
□ 1 □ 2 □ 3 □ 4 □ 5	9. Does it describe how each treatment works?
□ 1 □ 2 □ 3 □ 4 □ 5	10. Does it describe the benefits of each treatment?
□ 1 □ 2 □ 3 □ 4 □ 5	11. Does it describe the risks of each treatment?
□ 1 □ 2 □ 3 □ 4 □ 5	12. Does it describe what would happen if no treatment is used?
□ 1 □ 2 □ 3 □ 4 □ 5	13. Does it describe how the treatment choices affect overall quality of life?
□ 1 □ 2 □ 3 □ 4 □ 5	14. Is it clear that there may be more than 1 possible treatment choice?
□ 1 □ 2 □ 3 □ 4 □ 5	15. Does it provide support for shared decision making?
**Section 2 scores: _______**	
	**Section 3 Overall rating of the publication**
□ 1 □ 2 □ 3 □ 4 □ 5	16. Based on the answers to all of the above questions, rate the overall quality of the publication as a source of information about treatment choices:
	Low (1-2): Serious or extensive shortcomings
	Moderate (3-4): Potentially important but not serious shortcomings
	High (5): Minimal shortcomings
**Section 3 scores: ______**	
**DISCERN total scores:**	
